# Pyruvate-depleting conditions induce biofilm dispersion and enhance the efficacy of antibiotics in killing biofilms ***in vitr****o* and ***in vivo***

**DOI:** 10.1038/s41598-019-40378-z

**Published:** 2019-03-06

**Authors:** James Goodwine, Joel Gil, Amber Doiron, Jose Valdes, Michael Solis, Alex Higa, Stephen Davis, Karin Sauer

**Affiliations:** 1 Department of Biological Sciences, Binghamton Biofilm Research Center, Binghamton University, Binghamton, NY 13902 USA; 2 Department of Dermatology & Cutaneous Surgery, University of Miami Miller School of Medicine, Miami, FL 33136 USA; 3 Department of Biomedical Engineering, Binghamton Biofilm Research Center, Binghamton University, Binghamton, NY 13902 USA

## Abstract

The formation of biofilms is a developmental process initiated by planktonic cells transitioning to the surface, which comes full circle when cells disperse from the biofilm and transition to the planktonic mode of growth. Considering that pyruvate has been previously demonstrated to be required for the formation of *P. aeruginosa* biofilms, we asked whether pyruvate likewise contributes to the maintenance of the biofilm structure, with depletion of pyruvate resulting in dispersion. Here, we demonstrate that the enzymatic depletion of pyruvate coincided with the dispersion of established biofilms by *S. aureus* and laboratory and clinical *P. aeruginosa* isolates. The dispersion response was dependent on pyruvate fermentation pathway components but independent of proteins previously described to contribute to *P. aeruginosa* biofilm dispersion. Using porcine second-degree burn wounds infected with *P. aeruginosa* biofilm cells, we furthermore demonstrated that pyruvate depletion resulted in a reduction of biofilm biomass *in vivo*. Pyruvate-depleting conditions enhanced the efficacy of tobramycin killing of the resident wound biofilms by up to 5-logs. Our findings strongly suggest the management of pyruvate availability to be a promising strategy to combat biofilm-related infections by two principal pathogens associated with wound and cystic fibrosis lung infections.

## Introduction

Biofilms are defined as a structured community of bacterial cells enclosed in a self-produced polymeric matrix and adherent to inert or living surfaces^1^. The ability to form a biofilm is a common trait of a diverse array of microbes, including lower order eukaryotes, with biofilms having been recognized as the dominant mode of bacterial growth^1–3^. Biofilms are the root cause of a variety of chronic infections such as lung infections in patients suffering from cystic fibrosis and infections related to indwelling medical devices such as ventilator-associated pneumonia in intubated patients, and urinary tract infection due to catheters^1^. The National Institutes of Health estimated that 65% of all nosocomial infections are due to bacteria growing as biofilms and that biofilms are responsible for over 80% of microbial infections. Furthermore, the United States Center for Disease Control (CDC) estimated that biofilms are the etiologic agent in 60% of all chronic infections, including biofilms associated with cystic fibrosis (CF), burn wounds, and chronic wounds. Biofilm-related infections likely account for 1.7 million infections and 99,000 associated deaths, with over $7 billion spent annually on the treatment of nosocomial infections. The high infection and death toll has been attributed to the extraordinary resistance of biofilms to antimicrobial agents^1,4,5^, with biofilms having been reported to be up to 1000-fold less susceptible than their planktonic counterparts to various antibiotics^6^. In addition, biofilms have been reported to inhibit wound healing^7–9^. It is, thus, not surprising that biofilms have been reported to be refractory to conventional antibiotic treatment therapies^1,4^. For instance, while the development of antimicrobial burn creams was considered a major advance in the care of wound patients^10^, infections of wounds remain the most common cause of morbidity and mortality among the 6.5 million people suffering from wounds in the United States alone, causing over 200,000 deaths annually^11,12^. The most affected are people suffering from burn wounds, for which almost 61% of deaths are caused by infection^10,13^. Treatment failure has been primarily attributed to the principal wound pathogens *Staphylococcus aureus* and *Pseudomonas aeruginosa* and their ability to form biofilms in wounds^1,4,5,10^.

In the laboratory, biofilm formation is a cyclical process wherein a free-swimming planktonic cell encounters a surface – biotic or abiotic – and initiates cell-to-surface attachment. In *P. aeruginosa*, the process is highly-regulated and involves multiple phenotypically-distinct developmental stages^14–17^. This stage-specific developmental cycle was first recognized by Davies^18^ in 1996, and was further characterized in *P. aeruginosa* as a five-stage process by Sauer *et al*.^14^, consisting of reversible attachment, irreversible attachment, maturation-1, maturation-2, and dispersion. The last stage, dispersion, is a regulated process by which biofilm bacteria escape the sessile mode of growth as a means of self-preservation, and transition to the planktonic, free-living mode of growth^14,17^. The last stage furthermore provides a mechanism to enable dissemination to new locales for colonization.^14,17,19^. Dispersion coincides with 80% or more of the biofilm biomass being removed^20^, with the evacuating bacteria having been shown to be less protected from the immune system, and more susceptible to antimicrobial agents relative to the biofilms from which they evacuated (reviewed in^21,22^). Overall, dispersed cells have been reported to be as susceptible as planktonic cells (and biofilms at the reversible attachment stage) to antimicrobial agents compared to biofilm cells at other stages of development^23–25^. The finding suggested that transition to and from the surface coincides with major changes in the recalcitrance of bacterial cells to antimicrobial agents and immune cells^23,26^. It is thus, not surprising that manipulation of the biofilm developmental life cycle, attachment and dispersion, has been suggested to be a promising avenue open for biofilm control. In response, there have been numerous reports on strategies aiming at either preventing biofilms from forming or inducing biofilms to disperse as well as agents inducing the transition from a sessile to a planktonic (and *vice versa*, reviewed in^21^). For instance, dispersion can be induced upon sensing of self-synthesized signaling molecules. The factor responsible for native dispersion in *P. aeruginosa* biofilms has been identified as cis-2-decenoic acid (cis-DA), a fatty acid signaling molecule belonging to the family of diffusible signaling factors^27,28^. Exogenous addition of cis-DA also induces the dispersion of a large number of biofilm-forming bacteria including *P. aeruginosa, Escherichia coli, Klebsiella pneumoniae, Proteus mirabilis, Streptococcus pyogenes, Bacillus subtilis, Staphylococcus aureus*, and the yeast *Candida albicans*^28^. Likewise, dispersion can be induced by sensing of external conditions including starvation, oxidative or nitrosative stress, host factors such as bile salts, and availability of oxygen, iron, and carbon sources^29–35^. Several dispersion cues have been shown to also prevent biofilm formation, further supporting the notion that dispersion of biofilms is a reversion to the planktonic mode of growth. In the case of nitric oxide (NO), low levels of exogenous NO via the donor molecule sodium nitroprusside (SNP) have not only been shown to induce dispersion but also to prevent biofilm formation^32,36^. However, higher concentrations of NO in the millimolar range promoted the biofilm mode of growth, presumably due to enhanced anaerobic metabolism occurring at more acidic pH or due to adaptive responses from the biofilm bacteria^36^. Likewise, addition of the dispersion cue cis-DA to the growth medium prevents biofilm formation^28,37^. Additional factors regulating the formation of biofilms include quorum sensing molecules such as N-acylhomoserine lactone, farnesol, and indole^38–42^, with reports furthermore supporting a role of quorum sensing molecules in dispersion^43^.

Additional factors affecting biofilm formation are monocarboxylic acids such as pyruvate and lactate, the major product of pyruvate fermentation^44,45^. Similar to NO, pyruvate has been shown to impair or enhance biofilm formation in a concentration-specific manner, as addition of 10 mM pyruvate enhanced biofilm formation by *P. aeruginosa* while continuous depletion of pyruvate (via pyruvate dehydrogenase, PDH) from the growth medium prevented biofilm formation^45^. Pyruvate was found to be required to cope with stressful, oxygen**-**limiting but electron**-**rich conditions^45^, referred to as ‘reductive stress’ (too much NADH/electrons, not enough O_2_) present in biofilms. This is apparent by the activation of pyruvate fermentation pathways in biofilms, and mutant strains inactivated in genes involved in pyruvate fermentation, including *acnA* and *ldhA* encoding aconitase and lactate dehydrogenase, respectively, being unable to form biofilms^45^. Given the role of pyruvate in coping with the stressful, oxygen**-**limiting but electron**-**rich conditions that are prevalent in established biofilms, and that factors affecting the formation of biofilms have also been shown to induce dispersion, we asked whether pyruvate-depleting conditions not only prevent biofilms from forming but also induce biofilms to disperse.

## Results

### Depletion of pyruvate coincides with a reduction in the biofilm biomass

**Figure 1:**
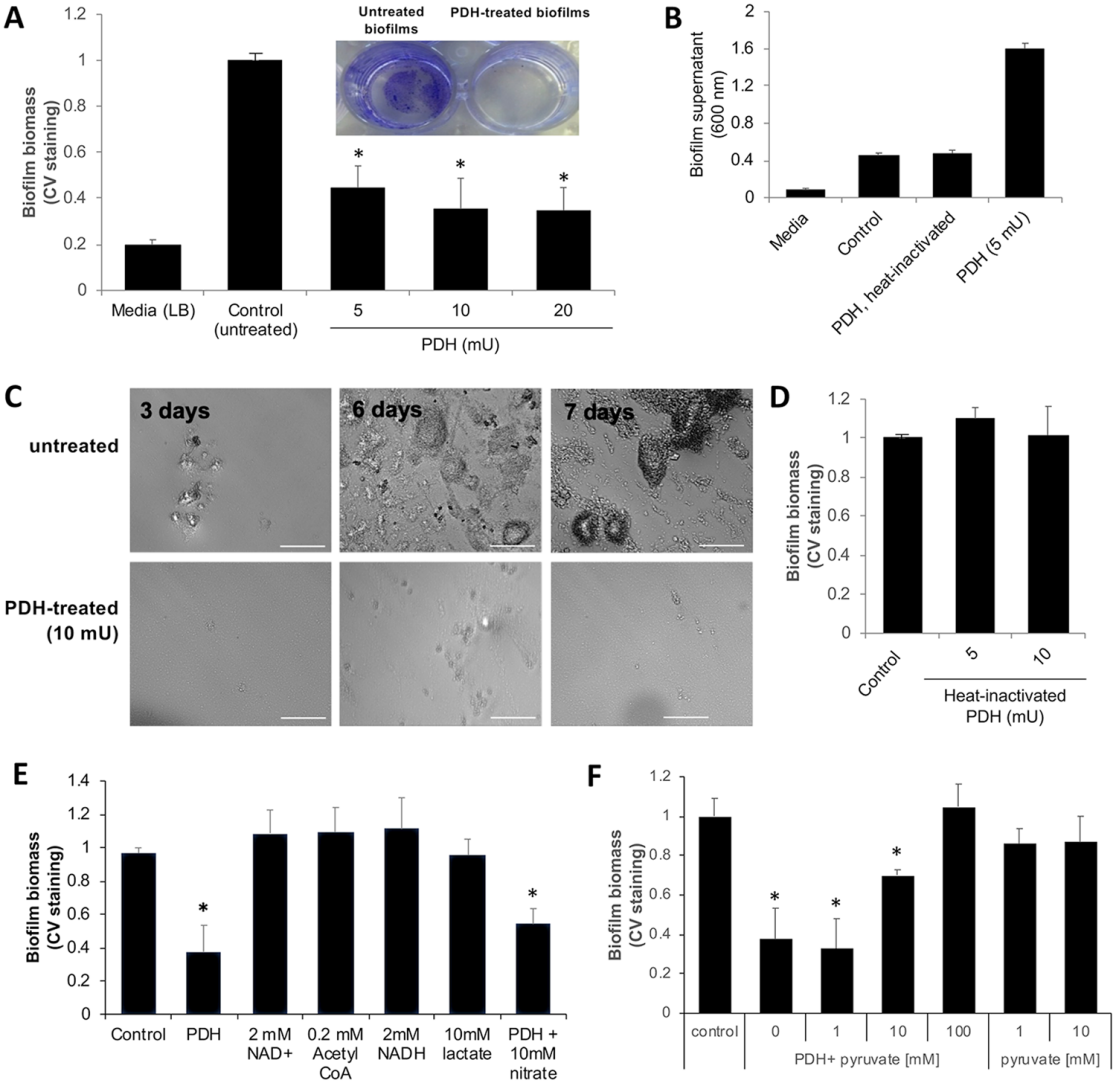
Exposure of *P. aeruginosa* biofilms to active pyruvate dehydrogenase (PDH) coincides with a reduction in the biofilm biomass in a manner independent of biofilm age. Biofilms were grown for 4 days in 24-well polystyrene plates in five-fold diluted LB. (**A**) Remaining biofilm biomass following exposure to 5, 10, and 20 mU PDH, as determined using CV staining. Inset, CV-stained biofilms prior to and post treatment with PDH. (**B**) Absorbance of biofilm supernatant following exposure to PDH or heat-inactivated PDH. (**C**) Brightfield images of biofilms grown for 3, 6, and 7 days prior to and post treatment with 10 mU PDH. (**D**) Biofilm biomass following exposure to heat-inactivated PDH. Remaining biofilm biomass following exposure to (**E**) 10 mM lactate or cofactors and products of the PDH catalyzed reaction, namely ß-NAD^+^ and acetyl-CoA, ß-NADH, and 10 mM nitrate, or to (**F**) increasing concentration of pyruvate (0, 1, 10, and 100 mM) in the presence and absence of PDH. Untreated biofilms were used as controls. PDH treatment was done in the presence of CoA, ß-NAD^+^, TPP, and MgSO_4._ *Significantly different (p < 0.05) from untreated biofilms. All experiments were carried out in triplicate. Error bars denote standard deviation.

To address the question whether depletion of pyruvate not only prevents the formation of biofilms by *P. aeruginosa* but also eliminates existing biofilms, *P. aeruginosa* biofilms grown for 4 days in 24-well plates were exposed to pyruvate-depleting conditions. To induce pyruvate depleting conditions, we made use of the enzyme pyruvate dehydrogenase (PDH) that catalyzes the conversion of pyruvate to acetyl-CoA in the presence of CoA and NAD^+^. Biofilms were exposed to increasing concentrations of PDH having a specific activity of 0.57U/mg. Specifically, biofilms were exposed to 5, 10, and 20 mU (8.7, 17.4, and 32.8 mg enzyme) of PDH in the presence of NAD^+^ and CoA. Biofilms grown in LB but left untreated were used as controls. Following overnight incubation, the remaining biofilm biomass was stained using crystal violet (CV). Relative to untreated biofilms, PDH treatment coincided with a significant loss in the CV-stainable biofilm biomass, with exposure to 5 mU resulting in a 2.2-fold reduction in the biofilm biomass while exposure to 10 and 20 mU resulted on average in a 2.9-fold reduction (Fig. 1A). The decrease in the biofilm biomass upon PDH treatment coincided with an increase in the absorbance of biofilm supernatants (Fig. 1B). Increased absorbance post PDH treatment coincided with an increase in the viable cells present in the supernatant from 1 × 10^8^ to 2 × 10^9^ CFU/ml.

To determine whether biofilms of different age are susceptible to pyruvate depleting conditions, we exposed biofilms grown for 2, 5, and 6 days to pyruvate depleting conditions, by treating biofilms with 10 mU PDH in the presence of cofactors. Following incubation for 16 h, microscopic evaluation of the remaining biofilms indicated exposure of biofilms to PDH to coincide with a significant reduction in the biofilm biomass relative to the biofilms that were left untreated (Fig. 1C).

Our findings suggested that exposure to PDH contributes to the loss of biofilm biomass regardless of the biofilm age. This was further supported by the finding that exposure of biofilms to increasing concentrations of heat-inactivated PDH had no effect on the biofilm biomass relative to untreated biofilms (Fig. 1D). Likewise, no increase in turbidity of the biofilm supernatant was noted (Fig. 1B).

### Pyruvate-depletion induced biofilm biomass loss is inhibited by pyruvate but not acetyl-CoA or lactate

To ensure that PDH induces a loss of the biofilm biomass by inducing pyruvate-depleting conditions, we furthermore thought to determine the effect of cofactors on the biofilm biomass. PDH requires NAD^+^ and CoA as cofactors to enzymatically convert pyruvate to acetyl-CoA and NADH. Exposure of biofilms to NAD^+^ and CoA or NAD^+^ alone had no effect on the biofilm biomass accumulation (Fig. 1E). We likewise determined whether the loss of the biofilm biomass upon exposure to PDH is due to the accumulation of the end products of the PDH catalyzed reaction, acetyl-CoA and NADH. Analysis of the biofilm biomass relative to untreated biofilms indicated that exposure of biofilms to 0.2 mM acetyl-CoA or 2 mM NADH did not result in increased biofilm biomass accumulation (Fig. 1E). We also tested lactic acid, a structurally similar monocarboxylic acid that is a product of pyruvate fermentation. Relative to untreated biofilms, however, no significant difference in the biofilms following exposure to 10 mM lactate was noted (Fig. 1E). It is of interest to note that the addition of nitrate had no negative effect on PDH inducing a dispersion response (Fig. 1E).

We also reasoned that if PDH induces dispersion by depleting pyruvate, the presence of additional pyruvate would overwhelm PDH, thus reducing the efficacy of PDH in inducing loss of biofilm biomass. We therefore exposed biofilms to increasing concentrations of exogenously added pyruvate (1–100 mM), in the presence of 10 mM PDH and cofactors. Relative to untreated biofilms, treatment with PDH in the presence of 1 and 10 mM pyruvate coincided with significantly reduced CV-stainable biofilm biomass (Fig. 1F). However, while no difference in the fold reduction of the biofilm biomass was noted in the presence of 1 mM pyruvate, the biofilm biomass was reduced by less than 2-fold in the presence of 10 mM pyruvate. Addition of 100 mM pyruvate completely abolished any difference in the biofilm biomass relative to untreated biofilms (Fig. 1F). Considering that pyruvate depleting conditions had no effect on the growth of *P. aeruginosa* under planktonic growth conditions^45^, our findings strongly suggest PDH to reduce the biofilm biomass in a manner dependent on pyruvate, further indicating that exposure of PDH induces biofilm biomass loss by depleting pyruvate.

### Depletion of pyruvate coincides with dispersion events

**Figure 2:**
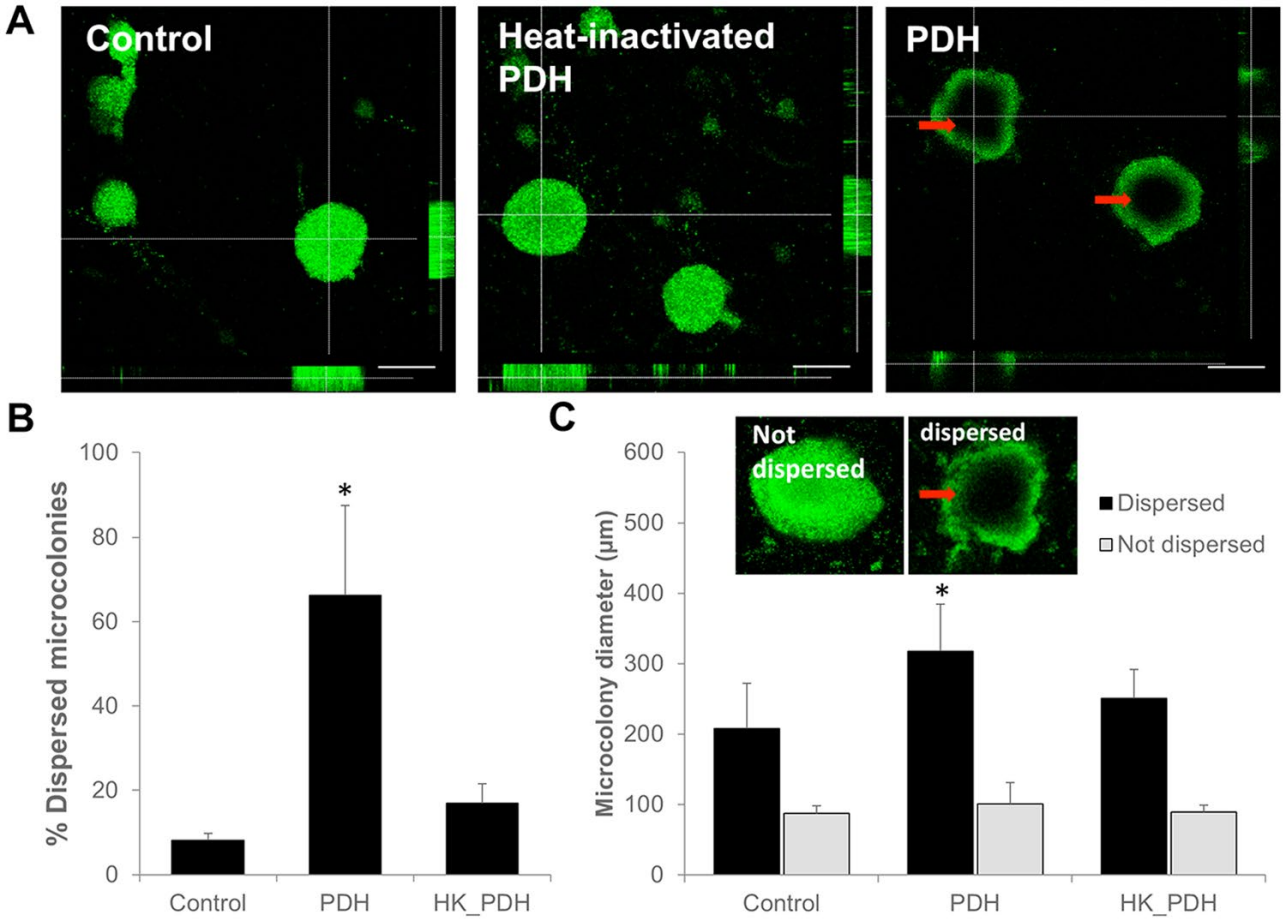
Pyruvate depletion coincides with *P. aeruginosa* biofilms showing signs of dispersion. (**A**) Representative confocal images of biofilms grown for 4 days in 24-well polystyrene plates that were left untreated (control), or were exposed to 10 mU PDH or 10 mU heat-inactivated PDH (HK_PDH). Red arrow indicates central hollowing of microcolony architecture. White size bar = 100 µm. (**B**) Number of microcolonies as percent of the total colonies counted per treatment group, showing void formation indicative of dispersion. (**C**) Average diameter of microcolonies that appeared to be dispersed or not dispersed. Inset, representative images of microcolonies that were considered in the analysis as “not dispersed” or “dispersed”. Error bars represent standard deviation of biological replicates. *Statistically different (p < 0.05) from untreated biofilms.

Our findings suggested exposure to PDH and thus, pyruvate-depleting conditions to coincide with significantly reduced biofilm biomass accumulation (Fig. 1A–C). To determine how exposure to PDH accomplished a reduction in the biofilm biomass, the remaining biofilm architecture was visually analyzed by confocal microscopy. Relative to untreated biofilms, biofilms exposed to PDH were not only characterized by an overall reduced biofilm biomass but by microcolonies having central voids (Fig. 2A). Void formation has been previously linked with native biofilm dispersion, the last stage of biofilm development in which sessile, surface-attached organisms liberate themselves from the biofilm to return to the planktonic state^14,17^. Overall, more than 60% of the detectable microcolonies present in PDH-treated biofilms showed signs of dispersion apparent by central voids (Fig. 2B). In contrast, the vast majority of microcolonies by untreated biofilms were intact (Fig. 2A), with only less than 8% of the microcolonies featuring central void formation (Fig. 2B). Similar results were obtained when biofilms were treated with heat-inactivated PDH (Fig. 2A,B).

Previous findings suggested microcolonies of *P. aeruginosa* to undergo dispersion events and form hollow voids at their center when they attain a minimum diameter of 40 µm, with the microcolony size within which these voids form being dependent on the fluid flow rate^27^. Given that exposure to PDH was found to coincide with a larger percentage of microcolonies showing void formation, we next asked whether pyruvate-depleting conditions affect the minimum diameter of microcolonies that disperse. Analysis of the microcolony size in untreated biofilms suggested that microcolonies with an average diameter of 90 µm were non-dispersed while larger microcolonies having an average size of 210 µm showed signs of dispersion (Fig. 2C). Exposure of biofilms to heat-inactivated PDH had little to no effect on the microcolony size of dispersed and non-dispersed microcolonies compared to control (Fig. 2C). Moreover, no significant difference in the size of non-dispersed microcolonies following exposure to PDH was noted. In contrast, however, we noticed an overall increase in the size of dispersed microcolonies of PDH treated biofilms (Fig. 2C). Based on visual observations, the increase in the size of dispersed microcolonies was likely due to “sagging”, with the remaining microcolony structure bulging downward under the weight, or pressure, or through lack of strength.

Overall, our findings suggest exposure of biofilms to PDH and thus, pyruvate depleting conditions, to coincide with dispersion events that were similar to events previously noted for native dispersion of biofilms. Considering that PDH treatment coincides with an overall increase in dispersion events without affecting the overall size of microcolonies that remain intact, our findings furthermore suggest that pyruvate depletion enhances dispersion.

### Pyruvate-depletion induced dispersion is dependent on lactate dehydrogenase LdhA and the microcolony formation regulator MifR

**Figure 3:**
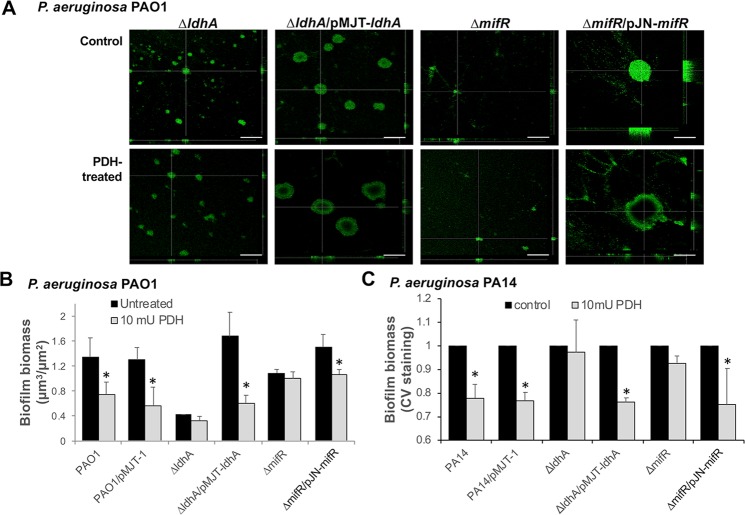
Pyruvate-depletion induced dispersion is dependent on MifR and functional *ldhA*. (**A**,**B**) Biofilms by indicated *P. aeruginosa* PAO1 mutant strains grown for 4 days in 24-well polystyrene plates that were left untreated or exposure to 10 mU PDH and cofactors for 16 h. (**A**) Representative confocal images and (**B**) quantitative analysis of the biofilm biomass by *P. aeruginosa* PAO1 and indicated mutant strains. (**C**) Remaining biofilm biomass of *P. aeruginosa* PA14 and indicated mutant strains that were either left untreated or exposure to 10 mU PDH and cofactors for 16 h, as determined using CV staining. White size bar = 100 µm. *Statistically different from untreated biofilms (p < 0.05). All experiments were carried out at least in triplicate. Error bars denote standard deviation.

We previously demonstrated that the formation of biofilms by *P. aeruginosa* requires pyruvate and pyruvate fermentation, with the biofilm-dependent utilization of pyruvate requiring lactate dehydrogenase LdhA and the microcolony formation regulator MifR^45^. Our findings furthermore demonstrated that biofilm formation is associated with stressful, oxygen-limiting but electron-rich conditions, suggesting pyruvate to be required to cope with such conditions^45^, referred to as ‘reductive stress’ (too much NADH/electrons, not enough O_2_) present in biofilms. We therefore asked whether the factors contributing to the formation of biofilms by *P. aeruginosa* also play a role in dispersion. Biofilms by mutant strains ∆*ldhA* and ∆*mifR* were exposed to PDH, and the biofilm structure of the respective mutant strains exposed to PDH relative to untreated biofilms were analyzed by confocal microscopy. Based on visual comparison, no difference in the biofilm architecture by ∆*ldhA* and ∆*mifR* in the presence or absence of PDH was noted (Fig. 3A). Quantitative COMSTAT analysis of the biofilm structure obtained in the absence/presence of PDH confirmed our observations of reduced biofilm biomass in response to PDH by wild-type biofilms but not ∆*ldhA* and ∆*mifR* biofilms **(**Fig. 3B**)**. Dispersion in response to pyruvate-depleting conditions, however, was restored upon complementation, apparent in biofilms by the complemented strains ∆*ldhA*/pMJT*-ldhA* and ∆*mifR*/pJN-*mifR* demonstrating voids upon exposure to PDH (Fig. 3A,B).

The findings of inactivation of *ldhA* and *mifR* impairing the pyruvate-depletion induced dispersion response by *P. aeruginosa* PAO1 were confirmed in *P. aeruginosa* PA14 strain using CV staining. While biofilms produced by *P. aeruginosa* PA14 dispersed in response to pyruvate-depleting conditions, no reduction in the biofilm biomass was noted upon exposure of biofilms by the mutant strains *ldhA::*IS and *mifR::*IS to PDH (Fig. 3C). Complementation of the *ldhA::*IS and *mifR::*IS mutant strains restored the dispersion response, apparent by the reduction in the CV-stainable biofilm biomass in a manner similar to the loss of biofilm biomass noted for *P. aeruginosa* PA14 (Fig. 3C).

Taken together, our findings strongly suggest exposure to PDH and thus, the pyruvate-depletion induced dispersion response, to require LdhA and MifR. Our findings furthermore suggest that dispersion induced by pyruvate depletion may be in response to biofilms no longer being capable of coping with the reductive stress^45^ present in biofilms.

### Pyruvate-depletion induced dispersion is independent of previously described factors contributing to dispersion

Considering that PDH-exposure of biofilms coincided with dispersion events, we next asked whether pyruvate-depletion induced dispersion requires factors previously demonstrated to be important in dispersion in response to nitric oxide or nutrients^30,46^. Specifically, we asked whether the chemotaxis transducer protein BdlA, and two phosphodiesterases, RbdA and DipA, play a role in the pyruvate-depletion induced dispersion response. The factors were chosen as they appear to play a central role in the dispersion response by *P. aeruginosa* biofilms, with inactivation of *bdlA, rbdA*, and *dipA* impairing the *P. aeruginosa* biofilm dispersion response upon exposure to a large number of dispersion cues including nutrients, NO, ammonium chloride, and heavy metals^30,32,47^.

**Figure 4:**
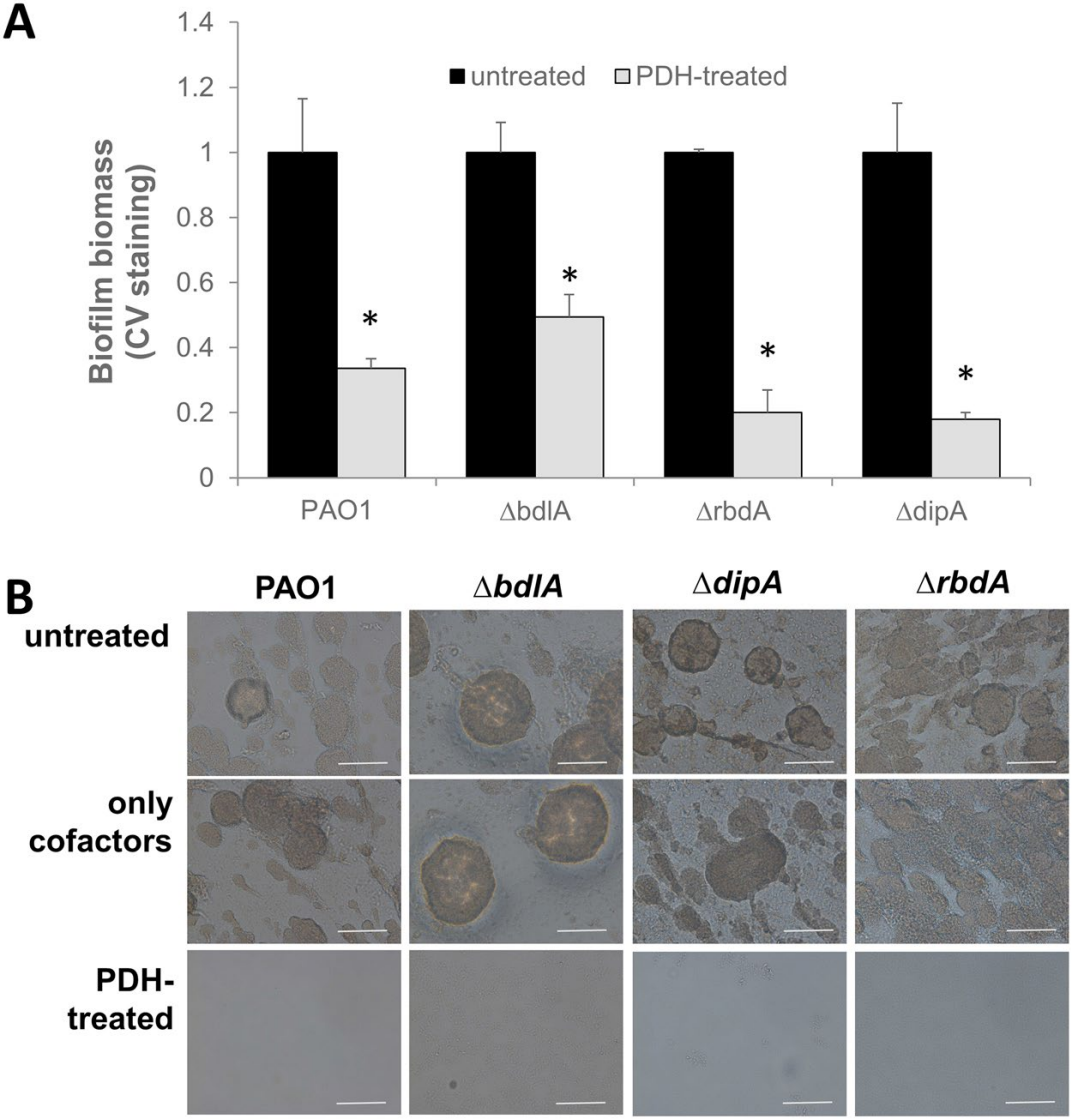
Pyruvate-depletion induced dispersion is independent of BdlA, DipA, and RbdA previously described to play a role in nutrient-induced dispersion. (**A**) CV-stained biomass and (**B**) representative brightfield images of biofilms by *P. aeruginosa* PAO1 and indicated mutant strains that either left untreated or exposure to 10 mU PDH and cofactors for 16 h. White size bar = 100 µm. *Statistically different from untreated biofilms (p < 0.05). All experiments were carried out at least in triplicate. Error bars denote standard deviation.

Biofilms by strains ∆*bdlA*, ∆*dipA*, and ∆*rbdA* were grown for 4 days, and subsequently exposed to 10 mU PDH to induce pyruvate-depleting conditions. Following overnight incubation, the remaining biofilm was stained using crystal violet. Relative to untreated biofilms, PDH treatment coincided with a significant loss in the CV-stainable biofilm biomass, with exposure of ∆*dipA*, and ∆*rbdA* biofilms to PDH resulting in a >60% loss of the biofilm biomass (Fig. 4A). Under the conditions analyzed, the reduction of the biofilm biomass was comparable to or exceeded the loss noted for wild-type biofilms (Fig. 4A). In contrast, the CV-stainable biomass by ∆*bdlA* biofilms was only reduced by 2-fold (Fig. 4A). However, the reduction in the biofilm biomass was significant. Our findings of PDH treatment resulting in reduced biofilm biomass by strains ∆*bdlA*, ∆*dipA*, and ∆*rbdA* were confirmed visually using brightfield microscopy (Fig. 4B).

### Pyruvate-depletion induced dispersion is not limited to the *P. aeruginosa* laboratory strain PAO1

**Figure 5:**
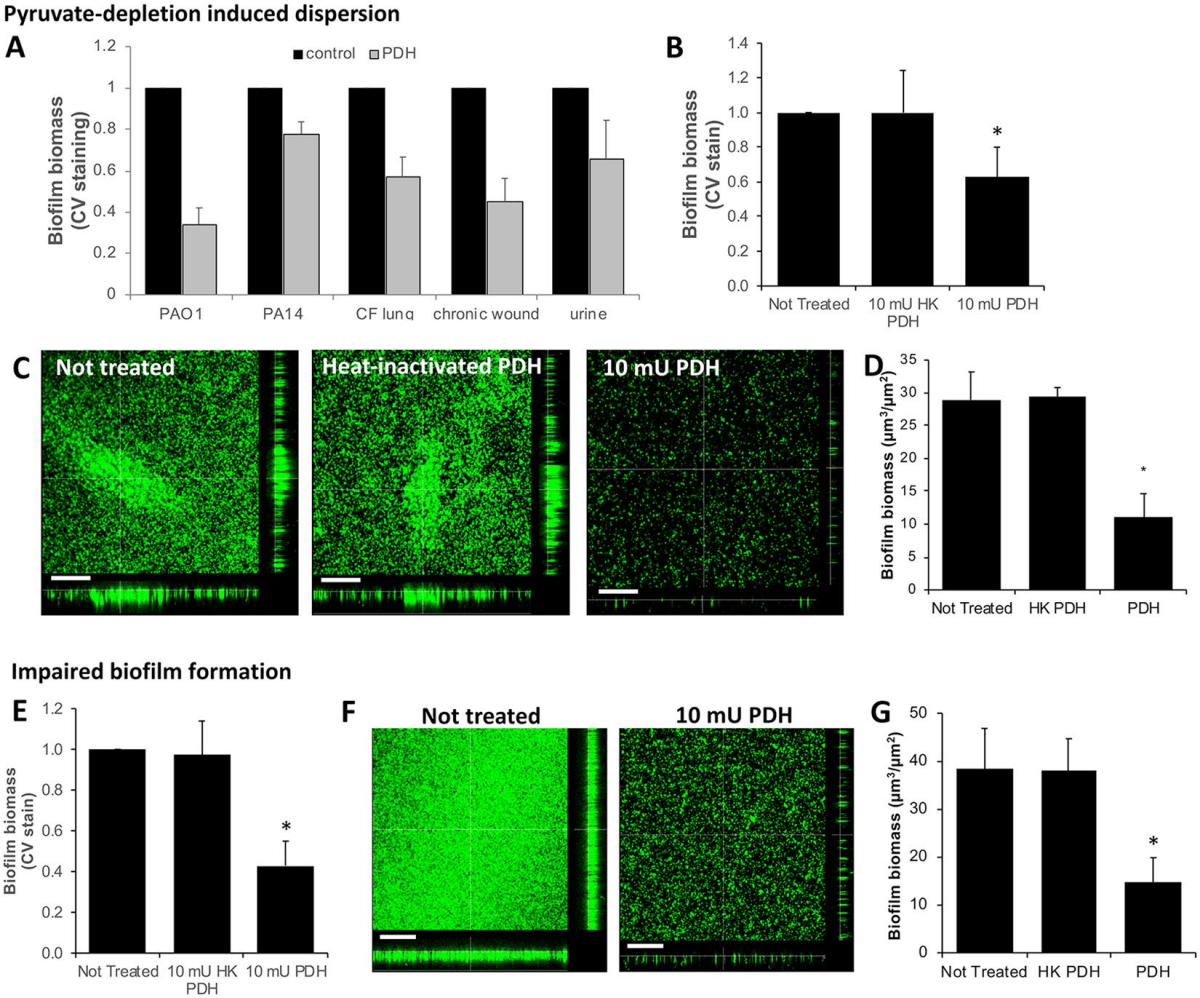
Effect of enzymatic depletion of pyruvate on biofilms by *P. aeruginosa* clinical isolates and *Staphylococcus aureus*. (**A**) Biofilm biomass of biofilms by *P. aeruginosa* strains PAO1, PA14, and clinical strains isolated from the cystic fibrosis lung, chronic wounds, and the urinary tract, left untreated or exposed to 10 mU PDH, as determined using CV staining. (**B**) Biofilm biomass of 4-day old *S. aureus* biofilms left untreated (control), and exposed for 16 h to 10 mU PDH or heat-inactivated PDH (HK_PDH), as determined using CV staining. (**C**) Representative confocal images of *S. aureus* biofilms left untreated (control), and exposed to 10 mU PDH or heat-inactivated PDH (HK_PDH). (**D**) COMSTAT analysis of the *S. aureus* biofilm biomass. **(E**–**G)** *S. aureus* biofilms were grown for 4 days in the absence (control) or continued presence of heat-inactivated PDH (HK PDH) or 10 mU PDH. (**E**) CV staining of remaining biofilm biomass following 4 days of growth. (**F**) Representative confocal images of *S. aureus* biofilms left untreated or continuously exposed to 10 mU PDH. (**G**) COMSTAT analysis of confocal of *S. aureus* remaining biofilm biomass. Size bars = 100 µm. All experiments were carried out at least in triplicate. Error bars represent standard deviation. *Significantly different from untreated biofilms (p < 0.05).

We furthermore asked whether dispersion in response to pyruvate depleting conditions is limited to the laboratory strains *P. aeruginosa* strain PAO1 and strain PA14. We therefore tested three *P. aeruginosa* clinical strains, isolated from various infection sites, including the urinary tract, burn wounds, and the lungs of cystic fibrosis patients. As shown in Fig. 5A, biofilms by these clinical isolates exhibited dispersion upon exposure to PDH (Fig. 5A). It is of interest to note, however, that the efficiency of PDH in reducing the biofilm biomass varied between 40–60%. The variability in the extent of the loss of CV-stainable biomass noted for clinical isolates, however, was within the range of the biofilm biomass loss noted for biofilms by the laboratory strains PAO1 and PA14 (Fig. 5A).

### Pyruvate-depletion inducing conditions affect the formation and stability of *Staphylococcus aureus* biofilms

To determine whether dispersion upon depletion of pyruvate is limited to biofilms by *P. aeruginosa*, we furthermore evaluated the effect of pyruvate depletion in *Staphylococcus aureus* ATCC 6538. We chose this facultative anaerobic, Gram-positive bacterium as *S. aureus* is a major cause of nosocomial and community-acquired infections and thus, represents a significant burden on the healthcare system^48^. Moreover, similar to *P*. *aeruginosa*, *S*. *aureus* is a common pathogen associated with lung infections of CF patients and chronic and burn wound infections^13,49,50^. Biofilms by 4-day old *S. aureus* were exposed to 10 mU PDH. Following overnight incubation for 16 h, the remaining biofilm was stained using crystal violet. Relative to untreated biofilms, PDH treatment coincided with a significant loss in the CV-stainable biofilm biomass, with exposure to 10 mU PDH resulting in a 40% reduction in the biomass of *S. aureus* biofilms (Fig. 5B). No reduction in the CV-stainable biofilm biomass was noted when biofilms were exposed to heat-inactivated PDH. The noted difference in CV staining in response to PDH treatment was confirmed by microscopic evaluation of the remaining biofilms. As shown in Fig. 5C, exposure of *S. aureus* biofilms to PDH and thus, pyruvate depleting conditions, coincided with a reduction in the biofilm biomass relative to the biofilms that were left untreated. Quantitative analysis of the biofilm structure by COMSTAT confirmed PDH treatment to coincide with a 40% reduction of the *S. aureus* biofilm biomass compared to the untreated biofilms and biofilms exposed to heat-inactivated PDH (Fig. 5D). Additionally, exposure to PDH coincided with decreased surface coverage, and a decrease in average and maximum thickness of *S. aureus* biofilms compared to untreated biofilms (not shown). Our findings strongly suggest that pyruvate depleting conditions result in the reduction of the biofilm biomass of established *S. aureus* biofilms. While pyruvate depleting conditions affected biofilms by *S. aureus*, pyruvate depleting conditions had no effect on the growth of *S. aureus* under planktonic growth conditions (not shown).

Given that enzymatic pyruvate depletion has been previously demonstrated to also prevent the formation of biofilms by *P. aeruginosa*, we furthermore asked whether pyruvate depleting conditions likewise impair the formation of *S. aureus* biofilms. To address this question, *S. aureus* biofilms were grown in the presence and absence of PDH. Following 4 days of growth, biofilms were stained using CV staining. Compared to untreated biofilms, continued exposure to PDH coincided with a significant, 2-fold reduction in the CV-stainable biofilm biomass (Fig. 5E). In contrast, no reduction was noted for biofilms exposed to heat-inactivated PDH relative to untreated biofilms (Fig. 5E). Confocal images acquired at the 4-day time point (Fig. 5F) and quantitative analysis of the biofilm structure confirmed that PDH treatment resulted in a significant reduction of the biofilm biomass relative to untreated biofilms and biofilms exposed to heat-inactivated PDH (Fig. 5F,G). Reduced biofilm biomass accumulation furthermore correlated with reduced surface coverage and reduced average and maximum thickness of biofilms (not shown).

Considering the visual observations combined with the quantitative CV and COMSTAT analysis, our findings suggest that like *P. aeruginosa*, both the formation and maintenance of the biofilm structure by *S. aureus* ATCC 6538 can be controlled by the availability of pyruvate.

### Pyruvate-depletion induced dispersion coincides with biofilms being rendered susceptible to tobramycin

**Figure 6:**
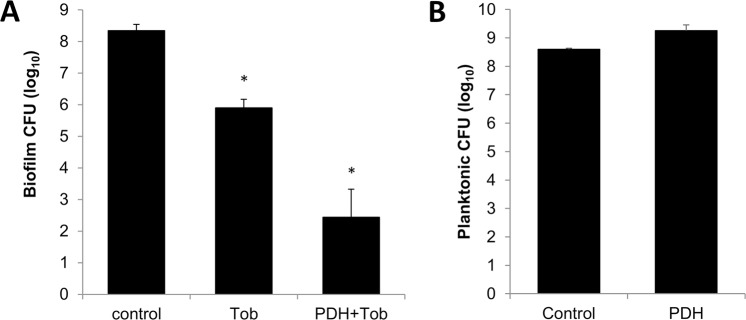
Pyruvate-depletion induced dispersion renders *P aeruginosa* biofilm cells more susceptible to the antibiotic tobramycin. (**A**) Viability (CFU) of *P. aeruginosa* PAO1 biofilms left untreated and exposed to tobramycin (150 µg/ml) or 10 mU PDH plus 150 µg/ml tobramycin for 1 h. (**B**) Viability (CFU) of *P. aeruginosa* PAO1 planktonic cells left untreated or exposed to 10 mU PDH for 1 h. Experiments were carried out at least in triplicate. Error bars represent standard deviation. *Significantly different (p < 0.05) from untreated biofilms.

It is now well established that biofilm and planktonic cells significantly differ in their drug susceptibility phenotype cells, with cells grown planktonically being significantly more susceptible to antimicrobial agents than cells grown in the biofilm mode of growth^21,26,51^. Likewise, dispersed cells have been reported to be more susceptible than biofilm cells, supporting the notion of dispersion being a reversion from the sessile to the planktonic mode of growth^21,25,52–54^. Considering that PDH treatment resulted in the dispersion of *P. aeruginosa* biofilms, we next asked whether treatment with dispersion-inducing PDH renders biofilms more susceptible to antimicrobial agents. We therefore exposed *P. aeruginosa* biofilms to 150 µg/ml tobramycin in the absence or presence of PDH. Following incubation, the viability of tobramycin-treated and control biofilms and biofilm supernatants was determined using viable cell counts. Relative to control biofilms, exposure of biofilms to tobramycin alone coincided in a 2.5-log reduction of the overall biofilm biomass, effectively reducing the number of viable cells from 2.4 × 10^8^ to 1.2 × 10^5^ cells per biofilm (Fig. 6A). Tobramycin treatment in the presence of PDH reduced the biofilm biomass to 3.5 × 10^3^ cells/biofilm (Fig. 6A), with the reduction in the viable cells being equivalent to an overall 5.9-log reduction in the biofilm biomass relative to untreated biofilms. Supernatants of control biofilms harbored on average 2 × 10^8^ CFU/well. In contrast, no viable cells were detected in supernatants of biofilms exposed to tobramycin or tobramycin plus PDH. It is of interest to note that exposure to PDH alone had no effect on the viability (Fig. 6B). Our findings clearly indicate that co-treatment with tobramycin in the presence of pyruvate-depleting conditions, rendered the antibiotic tobramycin more effective in killing biofilm cells. Overall, co-treatment enhanced the efficacy of tobramycin *in vitro* by 2.4-logs.

### Pyruvate-depletion reduces the biofilm burden in porcine burn wounds and enhances the efficacy of tobramycin in killing biofilm cells

Considering that PDH treatment coincided with dispersion and biofilms being rendered more susceptible to the antibiotic tobramycin relative to tobramycin alone, we next asked whether pyruvate depletion can be used as an anti-biofilm strategy *in vivo*, to reduce or eliminate biofilm-related infections.

Given that *P. aeruginosa* and *S. aureus* are considered the leading causes of biofilm infections, and are the principal pathogens associated with wound infections, we made use of a burn wound model of infection. We used a porcine rather than a rodent model, as pig skin is more similar to human skin, including similar epidermal/dermal-epidermal thickness ratios, dermal collagen, dermal elastic content, similar patterns of hair follicles, blood vessels, and physical and molecular responses to various growth factors^55–58^. Moreover, while rodent models heal primarily by contraction, pig skin heals in a manner similar to human skin by epithelialization^55,58,59^. It is thus not surprising that porcine wound models have become the standard tool for studying wound infections and testing new therapeutic agents^55–58^.

**Figure 7:**
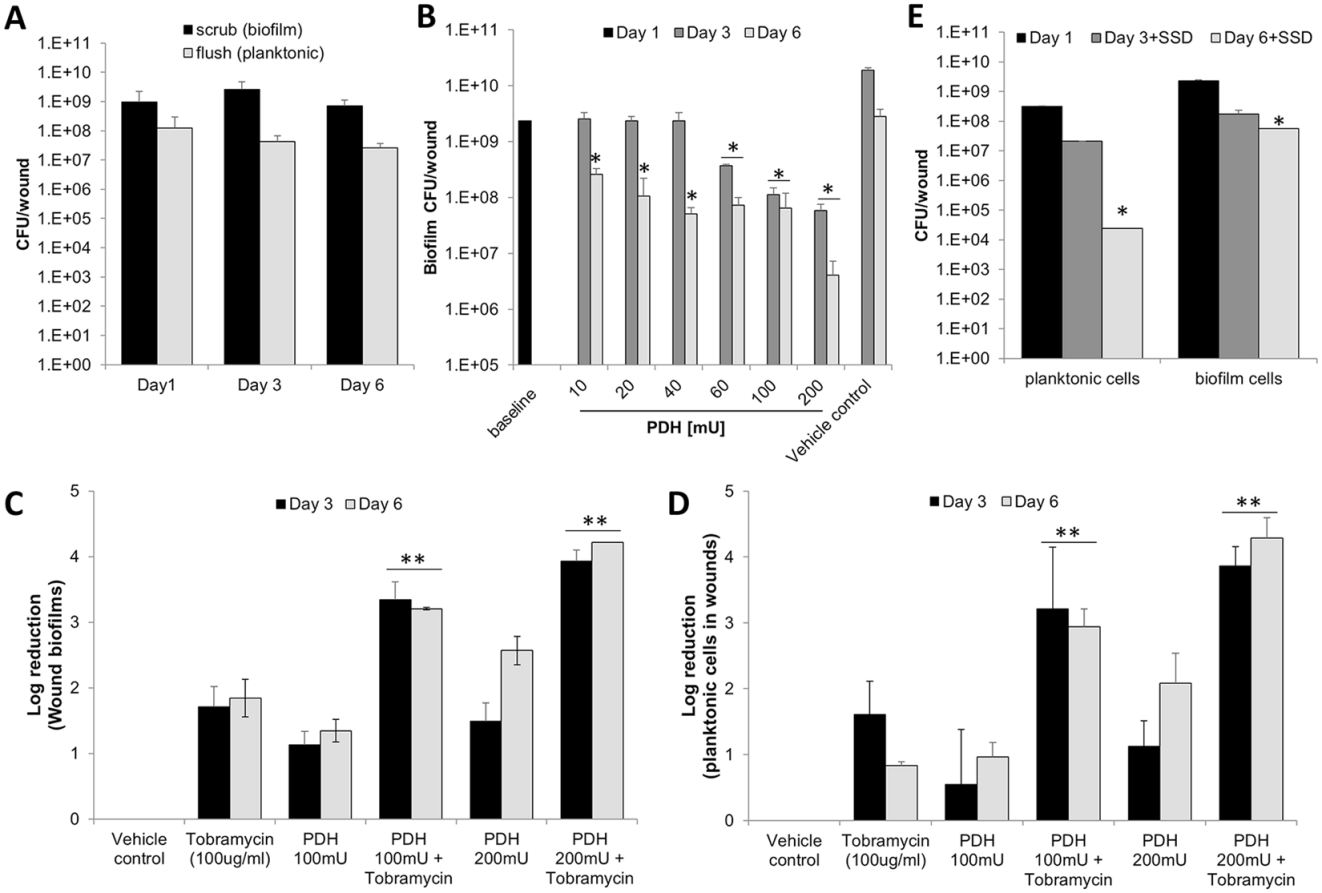
Pyruvate-depletion induced dispersion of *P. aeruginosa* biofilms within second-degree porcine burn wounds reduces the bacterial burden and enhances the efficacy of tobramycin in killing biofilm cells. Burn wounds were infected with *P. aeruginosa* ATCC® 27312™ and left untreated for 24 h. Wounds were subsequently exposed to increasing concentrations of PDH, tobramycin, or silver sulfadiazine. At days 1, 3 and 6 post infections, adherent and non-adherent bacterial cells were removed using flush & scrub, and the number of viable cells (CFU) per wound determined using viability counts. (**A**) Number of adherent (biofilm) and non-adherent (planktonic) cells present in wounds. (**B**) Effect of increasing concentrations of PDH on the *P. aeruginosa* biofilm population present in burn wounds. Efficacy of PDH (100 and 200 mU), tobramycin (100 µg/ml), or co-treatment of PDH and tobramycin on the (**C**) *P. aeruginosa* biofilm population and (**D**) planktonic population present in burn wounds. Log reduction was determined relative to untreated biofilms post 3 and 6 days infection. (**E**) Number of adherent (biofilm) and non-adherent (planktonic) cells present in wounds post exposure to silver sulfadiazine (SSD) 3 and 6 days post infection. Prior to SSD treatment, infected wounds were left untreated for 24 h post infection. With the exception of data shown in (**B**), all experiments were done in in triplicate, with each biological replicate being comprised of 3 wounds per treatment group (n = 9). Experiments shown in (**B**) are representative data obtained using only 3 wounds per treatment group (n = 3). Error bars represent standard deviation. Error bars represent standard deviation. *Significantly different (p < 0.05) from untreated wounds. **Significantly different (p < 0.05) from wounds treated only with PDH or tobramycin.

Burn wounds were infected with *P. aeruginosa* ATCC 27312. This strain was chosen as relative to the laboratory strain PAO1 and the wound clinical isolate, for several reasons. For one, strain ATCC 27312 is a well-known clinical burn wound isolate. In addition, strain *P. aeruginosa* ATCC 27312 was found to be more resistant to tobramycin, as determined using MIC assays (MIC of 0.8 µg/ml relative to 0.2 µg/ml for PAO1). Post infection, wounds were left untreated for 24 h to allow biofilms to establish. To confirm the formation of biofilms in wounds, bacterial cells present in untreated wounds were harvested using a flush& scrub technique^60,61^ that separates biofilm bacteria from planktonic bacteria, by flushing the non-adherent (planktonic) bacteria off the wound, followed by scrubbing of the wound to remove adherent biofilm-associated bacteria from the wound bed. Post 24 h of infection, wounds harbored 100-times more adherent cells than non-adherent cells, apparent by the flush & scrub method recovering on average 1 × 10^9^ biofilm cells and 1 × 10^8^ planktonic cells per wounds (Fig. 7A). Moreover, the bacterial population obtained by scrubbing remained constant post 3 and 6 days of infection (Fig. 7A). Our findings are in agreement with reports of adherent biofilm populations being established within 10–24 h post injury^62,63^.

To determine the optimal concentration of PDH needed to induce pyruvate-depleting conditions that result in loss of adherent biofilm cells and thus, dispersion, *in vivo*, we first exposed infected burn wounds with increasing concentrations of PDH ranging from 10–200 mU. While all tested PDH concentrations affected the bacterial burden present in wounds post 6 days of infection, daily exposure of infected wounds with 10–40 mU PDH had little to no effect on the biofilm population at 3 days post-infection relative to wounds at 1 day post-infection (Fig. 7B). Likewise, the vehicle control had no effect on the adherent biofilm population at any timepoints (Fig. 7B). Higher PDH concentrations, however, coincided with up to a 2-log reduction in the bacterial burden within 2 days of treatment, at 3 days post-infection (Fig. 7B). Our findings suggested PDH at concentrations of ≥ 60 mU are sufficient to reduce the biofilm burden *in vivo*. Moreover, it is of interest to note that no additional erythema (redness around the wound) indicative of prolonged inflammation was noted at any of the PDH concentrations tested (not shown).

Considering that our screen suggested pyruvate depleting conditions to result in a reduction of the biofilm burden in a manner similar to that noted under *in vitro* conditions, we next asked whether pyruvate-depleting conditions likewise enhance the efficacy of antibiotics in killing biofilms present in wounds. We therefore exposed wounds with established biofilms to a daily dose of 100 and 200 mU PDH in the absence or presence of 100 µg/ml tobramycin, and tobramycin alone. Relative to untreated wound biofilms, tobramycin treatment resulted in a reduction of the bacterial burden by approximately 2-log (Fig. 7C). Similar to tobramycin treatment alone, exposure to 100 mU and 200 mU PDH coincided with up to a 2-log reduction in the biofilm population present in wounds after 3 and 6 days of infection (Fig. 7C). Co-treatment with PDH and tobramycin coincided with an additional decrease in the adherent wound population. On average, co-treatment coincided with an approximate 2-log reduction relative to wounds exposed to PDH alone, and an approximate 4-log reduction relative to untreated biofilms (Fig. 7C). It is of interest to note that exposure to PDH alone and in combination with tobramycin likewise affected the non-adherent bacterial population present in wounds (Fig. 7D). Moreover, co-treatment with PDH and tobramycin coincided with an up to 4-log reduction in the non-adherent population in a manner similar to the adherent biofilm wound population (Fig. 7D).

We furthermore wanted to determine the efficacy of PDH relative to standard burn wound treatments. We therefore exposed infected wounds to silver sulfadiazine (SSD), a topical silver sulfonamide antibacterial agent, that has been the criterion standard for burn wound treatment for decades in the clinic^10^. Relative to untreated wounds, SSD treatment coincided with an approximate 4-log reduction in the planktonic population obtained by flushing, and reduction of the biofilm burden by approximately 2-log (Fig. 7E). The findings suggest that SSD is more efficient in reducing the non-adherent population in wounds relative to PDH alone, but reduction in the biofilm population is comparable. (Fig. 7C–E). Moreover, co-treatment with PDH and tobramycin exceeded the efficacy of SSD in reducing the biofilm population in wounds by 2-logs (Fig. 7C,E). It is of interest to note that we were unable to determine PDH in combination with SSD due to the denaturing effect of silver ions on the enzyme PDH.

## Discussion

Previous findings indicated pyruvate to contribute to growth of biofilms, with depletion of pyruvate or inactivation of lactate dehydrogenase impairing biofilm development and microcolony formation. The requirement of pyruvate fermentation in the initiation of biofilm formation suggested a role of pyruvate in redox balancing, to cope with stressful, oxygen-limiting but electron-rich conditions in biofilms^45^ referred to as reductive stress. Considering that established biofilms must cope with reductive stress in a similar manner to cells transitioning to the surface associated mode of growth, we asked whether pyruvate is required for the maintenance of the biofilm structure, with the enzymatic depletion of pyruvate from established biofilms resulting in biofilm cells reverting to the planktonic mode of growth. Here, we demonstrate that pyruvate depleting conditions coincided with loss of the *P. aeruginosa* biofilm biomass and that biofilm biomass loss was due to dispersion, indicative by void formation within the biofilm architecture. More specifically, our findings suggest dispersion to be due to impaired maintenance of redox balance and cellular homeostasis, induced by pyruvate deprivation. This is supported by *P. aeruginosa* having been demonstrated to produce and secrete pyruvate under favorable conditions but to utilize pyruvate under anoxic conditions to sustain survival^45,64,65^. Moreover, biofilms are characterized by steep oxygen and nutrient gradients, with cells located deep within biofilm structures experiencing stressful, growth-limiting conditions^66,67^. Microelectrode experiments have revealed that, while the concentration of oxygen at the surface of the biofilm is similar to that of the bulk fluid, oxygen levels drop rapidly towards the interior of biofilms with the center of microcolonies being essentially anoxic^68–70^. It is thus likely that *Pseudomonas aeruginosa* biofilm cells localized at the periphery and interstitial microaerophilic/anaerobic regions of biofilm microcolonies produce and secrete pyruvate that not only diffuses out into the surrounding environment but also toward the central anoxic core of microcolonies. Moreover, when biofilm microcolonies are small, pyruvate freely diffuse from the periphery of the biofilm to the center of the biofilm. However, when cell clusters attain a dimension where pyruvate no longer adequately reaches the interior (rate of consumption exceeds diffusion), pyruvate is no longer attained at a concentration necessary to sustain anoxic survival. Considering the similarity between pyruvate-depletion induced dispersion and native dispersion, our findings suggest native dispersion to be linked to redox balancing or reductive stress, likely induced by pyruvate limiting conditions within the biofilm.

Additional support of PDH treatment inducing dispersion stems from the finding of PDH-treated biofilms being rendered more susceptible to tobramycin than untreated biofilms (Fig. 6). Our findings are in agreement with reports of *P. aeruginosa* biofilms dispersed in response to cis-DA, nitric oxide and glutamate being likewise rendered more susceptible than non-dispersed biofilms^20,25,27,28,47,53,71^. However, the mechanism by which pyruvate depletion induces dispersion is distinct from previously described dispersion response in response to nitric oxide and carbon sources. This is supported by the finding that PDH-induced dispersion was impaired upon inactivation of MifR and lactate dehydrogenase (Fig. 3), but not upon inactivation of *bdlA, dipA*, and *rbdA* (Fig. 4). Despite the apparent differences, both mechanisms lead to biofilm biomass loss. Dispersion in response to nitric oxide and other dispersion inducing cues only coincides with up to 80% of the biofilm biomass dispersion from the biofilm. Likewise, PDH-induced dispersion was shown here not to result in the removal of the entire biofilm. Given the likely difference in mechanism, it is possible that pyruvate depleting conditions may be used in combination with other dispersion-inducing conditions to further augment the efficacy of the dispersion response. However, additional research will be required to determine if combination of such dispersion inducing conditions will indeed enhance the dispersion response.

We furthermore investigated the clinical applicability of utilizing PDH and pyruvate-depleting conditions in treating biofilm-related infections using an established chronic porcine burn wound model. We found that similar to our *in vitro* results, exposure of wound biofilms to PDH significantly reduced the biofilm biomass formed *in vivo*. Likewise, exposure to PDH coincided with a decrease in the planktonic population present in wounds. As planktonic cells have been reported to be more susceptible than their sessile counterparts to killing by the immune system, it is likely that PDH treatment may improve the ability of the host to eradicate recalcitrant biofilm infections. Our findings, however, are in contrast to reports by Fleming *et al*.^72^. Using the enzyme glycoside hydrolases to disaggregate biofilms in a mouse wound model, the authors noted large-scale dispersal events, apparent by dissemination of infection and possibly lethal septicemia in the absence of antibiotic therapy^72^. Moreover, treatment of biofilms grown *in vitro* and in the chronic porcine burn wound model with the antibiotic tobramycin displayed increased efficacy when combined with PDH. Likewise, co-treatment was found to be more effective than treatment with the antimicrobial cream silver sulfadiazine (SSD), which is considered the gold standard in wound care^10^. More specifically, while SSD only reduced the bacterial burden by *P. aeruginosa* biofilms by ~2 log CFU/wound (Fig. 7E), combination treatment of PDH and tobramycin resulted in a ≤5-log reduction in viability (Fig. 7C). Overall, our findings suggest that targeting pyruvate availability potentiates the efficacy of antimicrobial agents in killing biofilm cells and thus, improves the ability of both the host (and the clinician) to eradicate recalcitrant biofilm infections.

Taken together, our data indicate pyruvate to act as a switch to control biofilm formation, biofilm dispersion, and tolerance, with depletion of pyruvate coinciding with prevention of biofilm formation, disaggregation of existing biofilms, and dispersed biofilms being rendered susceptible to lower doses of antibiotics relative to intact biofilms both *in vitro* and *in vivo*. Our findings appear to be applicable to two pathogens, *P. aeruginosa* and *S. aureus*, that are responsible for over 54% of deaths of burn victims. Our findings furthermore support the notion of pyruvate depletion being an effective anti-biofilm therapy, capable of controlling and eradicating biofilms, by enhancing the efficacy of antibiotics and likely, the immune system. It should be noted that we used soluble PDH in the treatment of infected porcine burn wounds. Moreover, the PDH treatment regime used here made use relatively high concentrations to achieve significant dispersion, apparent by a detectable reduction in the biofilm biomass over a short period of time. That being said, we envision future PDH based anti-biofilm therapies to be based on a more controlled administration of the enzymes to the biofilm, using delivery media such as creams or extended release hydrogels. We not only envision such delivery vehicles to better retain PDH on the wounds during treatment but also to eventually protect PDH from proteolytic cleavage or the denaturing effect of silver ions present in SSD. In addition to PDH inducing dispersion and rendering antibiotics more effective in killing biofilm cells, a treatment strategy based on pyruvate depletion has several added benefits. In *P. aeruginosa*, pyruvate has only been linked to biofilm growth and long-term bacterial survival under oxygen limiting conditions, with long-term survival and biofilm studies not giving rise to pyruvate-insensitive mutants^45,64,65^. In agreement with previous findings, pyruvate depletion does not affect bacterial growth in liquid or susceptibility of planktonic cells^45^. All of the above lessen the possible selection of pyruvate-insensitive bacteria. Moreover, pyruvate depleting conditions impair the formation of biofilms, suggesting pyruvate management can also be used to prevent biofilm formation *in vivo*. Overall, our reported findings combined with PDH representing a likely effective anti-biofilm therapy, have the potential to impact areas of medical biofilm research beyond chronic wounds.

## Material and Methods

### Bacterial strains, plasmids, and culture conditions

The wild-type *Staphylococcus aureus* and *Pseudomonas aeruginosa* PAO1, isogenic mutants, and clinical isolates used in this study are listed in Table 1. Likewise, all plasmids are listed in Table 1. Bacterial overnight cultures by *P. aeruginosa* were grown at 37 °C in Lennox broth (LB) medium (BD Biosciences) in flasks with continuous shaking at 220 rpm while *S. aureus* was cultured in brain heart infusion medium (BHI, BD Biosciences). Plasmids were maintained by supplementing LB carbenicillin (250 μg/ml) and gentamicin (50 μg/ml) for growth of *P. aeruginosa*.

**Table 1:** Strains and plasmids.

Strains/plasmids	Relevant genotype or description	Source
	**Strains**	
*Pseudomonas aeruginosa*		
PAO1	Wild type	B.H. Holloway
∆*ldhA*	PAO1; PA0927::IS*lacZ*; Tet^R^	^81^
∆*mifR*	PAO1, ∆*mifR* (PA5511)	^45^
*∆bdlA*	PAO1, ∆*bdlA*	^47^
*∆dipA*	PAO1, ∆*dipA* (PA5017)	^46^
*∆rbdA*	PAO1, ∆*rbdA*	^46^
PA14	Wild type	^82^
∆*mifR::IS*	PA14 *mifR*::MAR2xT7; Gm^R^	^83^
∆*ldhA::IS*	PA14 *ldhA*::MAR2xT7; Gm^R^	^83^
*Pseudomonas aeruginosa* clinical isolates		
Chronic wounds	PA215; *P. aeruginosa* isolated from a chronic wound debridement samples from patients at Southwest Regional Wound Clinic (Lubbuk, TX)	^84, 85^
Cystic fibrosis	CF1–2; Classic *P. aeruginosa* isolate from newborn diagnosed with CF	^86^
ATCC 27312	Isolated from infected human wound infection; used for second-degree burn wound studies	ATCC
*Staphylococcus aureus*		
ATCC 6538	Wild type	ATCC
	**Plasmids**	
pRK2013	Helper plasmid for triparental mating; *mob*; *tra;* Km^R^	^87^
pJN105	Arabinose-inducible gene expression vector; pBRR-1 MCS; *araC-*P_*BAD*_; Gm^R^	^88^
pJN-*mifR*	*mifR*, amplified using PA5511_NheI_for/PA5511_EcoRI_rev, cloned int amplified using *ldhA*_NheI_for/ldhA_SacI_rev, cloned into pJN105, Gm^R^	^45^
pMJT-1	Arabinose-inducible gene expression vector; pUCP18 MCS; *araC-*P_*BAD*_; Amp/Carb^R^	^89^
pMJT-*ldhA*	*ldhA*, amplified using *ldhA*_NheI_for/ldhA_SacI_rev, cloned into pMJT-1, Amp/Carb^R^	^45^

### Biofilm growth

Biofilms were grown in a 24-well plate system modified from the procedure described by Caiazza and O’Toole^73^ to elucidate the role of pyruvate in dispersion. Each well has a growth area of 2 cm^2^ allowing for sufficient biofilm biomass development for microscopy, crystal violet staining, and susceptibility assays. Briefly, overnight cultures by *P. aeruginosa* were adjusted to an OD600 of 0.1 and 10 µl of the OD-adjusted suspension were added to wells of 24-well flat-bottom polystyrene microplates (BD Falcon™), with each well containing 250 µl 5-fold diluted LB medium. Biofilms were subsequently allowed to grow at 37 °C with while shaking at 220 rpm, with 24-well plates being kept at a 30° angle. The 30° angle promotes biofilm development by increasing the air-liquid interface. The medium was exchanged every 12 h for up to 5 days. *S. aureus* biofilms were grown similarly except that biofilms were grown in 250 µl of 5-fold diluted BHI in 24-well tissue culture plates (Greiner Bio-One) under static conditions.

### Pyruvate-depleting conditions

To determine whether pyruvate-depleting conditions induce biofilm dispersion, pyruvate was depleted from the growth medium of *P. aeruginosa* and *S. aureus* biofilm cultures through the enzymatic action of porcine-derived pyruvate dehydrogenase (PDH) (Sigma). The specific activity of PDH was 0.57 U/mg. As the enzyme was present in a storage solution composed of 50% glycerol solution containing ~9 mg/mL bovine serum albumin, 30% sucrose, 1.5 mM EDTA, 1.5 mM EGTA, 1.5 mM 2-mercaptoethanol, 0.3 TRITON® X-100, 0.003% sodium azide, and 15 mM potassium phosphate, pH 6.8, the storage solution was replaced with 50 mM MOPS/HCl pH 7.4 prior use. As a control, PDH was heat inactivated by boiling the enzyme for 10 min at 100 °C.

Briefly, biofilms were grown for up to 5 days prior to the addition of 1–20 mU PDH in the presence of 2 mM CoA (Sigma), 2 mM ß-NAD^+^ (Sigma), 20 µM thiamine pyrophosphate (TPP) (Sigma), and 50 µM magnesium sulfate (MgSO_4_) in 5-fold diluted LB or BHI. Biofilms were exposed to PDH plus cofactors for a total of 16 h. Where indicated, 1–10 mM pyruvate, 10 mM lactate, 1–20 mU pyruvate dehydrogenase (PDH), 2 mM β-NAD^+^, 2 mM β-NADH, or 2 mM sodium CoA was added. Heat-inactivated PDH in the presence of cofactors, or cofactors alone were used as controls. All experiments were performed in at least biological triplicate using 2–4 technical replicates.

To determine whether pyruvate-depleting conditions prevent biofilm formation by *S. aureus*, 10 mU PDH in the presence of 2 mM CoA, 2 mM ß-NAD^+^, 20 µM TPP, and 50 µM magnesium sulfate were added to the growth medium (5-fold diluted BHI) at the time of inoculation, subsequently replenished every 12 h. Heat-inactivated PDH in the presence of cofactors was used as control. All experiments were performed in biological triplicate using 4 technical replicates.

### Analysis of the biofilm structure

At indicated time points, the adherent cells were visualized by bright field or confocal laser scanning microscopy (CLSM) images using an Olympus BX60 microscope (Olympus, Melville, NY, USA) or a Leica TCS SP5 confocal microscope (Leica Microsystems, Wetzlar, Germany), respectively. For confocal image acquisition, biofilms were stained with the LIVE/DEAD BacLight Bacterial Viability Kit (Life Technologies). Quantitative analysis of the confocal laser scanning microscope images of 24-well plate-grown biofilms was performed using COMSTAT (7). Size determination of cellular aggregates and microcolonies was done using ImageJ^74^. At least ten separate images per treatment were counted, measured in diameter, and scored as dispersed or not dispersed based on central hollowing of microcolony architecture. Moreover, adherent cells were quantitated using crystal violet (CV) staining. Briefly, 50 μl CV stain was directly added to each well, followed by incubation for 15 min at 37 °C with continuous shaking at 220 rpm. Plates were washed three times with water, and allowed to dry prior to the addition of 200 μl ethanol to each well and subsequent incubation for 15 min at 37 °C with continuous shaking at 220 rpm. Finally, the OD_600nm_ was determined. Data were normalized to the values obtained for controls.

### Antimicrobial susceptibility assay

To determine whether PDH treatment of *P. aeruginosa* biofilms affected the susceptibility of biofilms to antimicrobial agents, biofilms were first allowed to grow in 24-well plates for 4 days, and then exposed to 10 mU PDH for 16 h. Then, tobramycin at a concentration of 150 µg/ml was added to the biofilms and incubated for an additional hour. Following the removal of the supernatants, biofilms were harvested by scraping into saline (0.85% NaCl), homogenized, diluted in saline, and drop-plated onto LB agar. Viable cell counts were analyzed following overnight incubation at 37 °C. All antibiotic susceptibility assays were performed in biological triplicate using technical duplicates. MIC assays were carried out as previously described^75^.

### Porcine second-degree burn wound model

The ability of PDH to disperse *P. aeruginosa* biofilms *in vivo* and enhance the efficacy of antimicrobial agents in killing biofilms was tested using a porcine burn wound model. Prior to initiation, the protocol was approved by the University of Miami’s IACUC. A total of three animals were used in this study, and second degree burn wounds were created on the back of the animals as previously described^76^. Burn wounds were inoculated with 25 µl of a standardized *P. aeruginosa* ATCC® 27312™ suspension harboring 10^6^ CFU/ml by depositing the aliquot into the center of each wound site^77–80^. The suspension was lightly scrubbed into the burn site for ten seconds using a sterile Teflon spatula. To determine the optimal treatment to disperse established biofilms, wounds were infected and covered post-infection under a polyurethane film for 24 h to promote biofilm formation^79^ and subsequently treated with increasing PDH concentrations (10–200 mU). PDH exposure to 100 mU and 200 mU was chosen for further study and co-treatment with 100 µg/ml tobramycin. Tobramycin treatment alone as well as untreated infected wounds were used as control. All wounds including control wounds were covered post-infection and/or PDH treatment with a polyurethane dressing to prevent any cross-contamination.

Three wounds were cultured 24 hours (Day 1) after wounding and inoculation for baseline enumeration of bacteria. Three wounds for each treatment group were recovered on Day 3 and 6 after inoculation using a flush & scrub technique. This specific harvesting technique separates out adherent bacteria from free floating, planktonic bacteria, by flushing the non-adherent (planktonic) bacteria off the wound, followed by scrubbing of the wound to remove the adhered bacteria (biofilm) from the wound bed. A sterile glass cylinder (22 mm outside diameter) was placed over the wound area. One ml of scrub solution was pipetted into the glass cylinder and the site was gently washed three times to remove the loosely attached bacteria. This aliquot, referred to as the flush culture representing the planktonic bacteria, was aspirated out and assessed for viable bacteria. The same wound was then encircled using another sterile glass cylinder and a sterile spatula was used to scrub the wounds for 30 seconds to remove the firmly attached or biofilm-associated bacteria. All culture samples were diluted and the extent of microbiological contamination was assessed using the Spiral Plater System (Spiral Biotech, Norwood, MA). This system deposits 50 μl of each flush or scrub bacterial suspension over the surface of a rotating agar plate. Pseudomonas Agar-base with CN supplement was used to isolate *P. aeruginosa* from the wounds. All plates were incubated aerobically overnight (24 h, 37 °C), after which the number of viable colonies (CFU) was counted and represented herein as a log_10_.

To ensure reproducible and statistically robust *in vivo* data, all experiments were carried out in triplicates, with each dataset being composed of 3 technical replicates (here, wounds) per assessment time per animal (total n = 9 per treatment group per assessment time).

### Ethics statement

All animal studies were approved by the University of Miami’s Institutional Animal Care and Use Committee (IACUC, protocol #17–117). All research involving animals was carried out in strict accordance with the Animal Welfare Act regulations overseen by the USDA, and the Public Health Service Policy on Humane Care and Use of Laboratory Animals (PHS Policy) administered by the National Institutes of Health, Office of Laboratory Animal Welfare.

### Statistical analysis

For multiple comparisons of means, a one-way analysis of variance (ANOVA) was used.
